# Narrative Review on the Effects of Oat and Sprouted Oat Components on Blood Pressure

**DOI:** 10.3390/nu14224772

**Published:** 2022-11-11

**Authors:** DeAnn J. Liska, ElHadji Dioum, Yifang Chu, Eunice Mah

**Affiliations:** 1 Consultant, Ridgefield, WA 98642, USA; 2 PepsiCo R&D Health & Nutrition Sciences, Chicago, IL 60607, USA; 3 Biofortis Research, Addison, IL 60101, USA

**Keywords:** Avena, diastolic, systolic, endothelial function, arterial, meta-analysis, phytonutrient, lifestyle, β-glucan

## Abstract

Hypertension (HTN) is a major risk factor for cardiovascular disease (CVD) and cognitive decline. Elevations in blood pressure (BP) leading to HTN can be found in young adults with increased prevalence as people age. Oats are known to decrease CVD risk via an established effect of β-glucan on the attenuation of blood cholesterol. Many past studies on CVD and oats have also reported a decrease in BP; however, a thorough assessment of oats and BP has not been conducted. Moreover, oats deliver several beneficial dietary components with putative beneficial effects on BP or endothelial function, such as β-glucan, γ-amino butyric acid (GABA), and phytochemicals such as avenanthramides. We conducted a comprehensive search for systematic reviews, meta-analyses, and clinical intervention studies on oats and BP and identified 18 randomized controlled trials (RCTs) and three meta-analyses that supported the role of oats in decreasing BP. Emerging data also suggest oat consumption may reduce the use of anti-hypertensive medications. The majority of these studies utilized whole oats or oat bran, which include a vast array of oat bioactives. Therefore, we also extensively reviewed the literature on these bioactives and their putative effect on BP-relevant mechanisms. The data suggest several oat components, such as GABA, as well as the delivery of high-quality plant protein and fermentable prebiotic fiber, may contribute to the anti-HTN effect of oats. In particular, GABA is enhanced in oat sprouts, which suggests this food may be particularly beneficial for healthy BP management.

## 1. Introduction

Blood pressure (BP) is an essential aspect of overall health and, when elevated, can lead to arterial damage and stroke [1,2,3]. Moreover, robust and consistent evidence indicates that elevated systolic blood pressure (SBP) and diastolic blood pressure (DBP) are associated with increased risk for cardiovascular disease (CVD) and end-stage renal disease [2,4]. High BP, which includes prehypertension and Stage 1 and Stage 2 hypertension (HTN), is a leading cause of death and morbidity worldwide and is also implicated in cognitive decline, which can occur as early as young adulthood with increased prevalence in older people [2,5,6]. The prevalence of HTN in older adults is substantial, and the 40-year risk of developing HTN after age 45 is estimated to be 93%, 92%, 86%, and 84% for African-Americans, Hispanics, white, and Chinese Americans, respectively [2]. A major concern with high BP is the prevalence of undiagnosed HTN due to the lack of noticeable symptoms, which is why it is called the silent killer [7].

Reducing SBP and DBP by 2 mmHg or 1 mmHg, respectively, is associated with a 10% reduction in the population risk of CVD, and a reduction of 5 mmHg is associated with reductions in stroke (34%) and coronary heart disease (21%) [8]. High BP is related to body weight, and every 1 kg of weight loss in people who are overweight or obese can decrease BP by around 1 mmHg [2,4]. Diet is an important factor as a primary intervention for prehypertension and an adjunct therapy when pharmacological interventions are warranted [2,7]. These include whole dietary modifications, such as the DASH diet, that are characterized by lower sodium and red meats and higher vegetables and low-fat dairy products [9] or supplementation with nutraceuticals such as potassium, magnesium, L-arginine, vitamin C, cocoa flavonoids, beetroot juice, coenzyme Q10, controlled-release melatonin, and aged garlic extract [10]. In particular, dietary fiber has received a great deal of attention for BP management since diets high in fiber are often promoted for weight loss/weight-loss maintenance and are associated with decreased risk of CVD and many other diseases [7,11]. Indeed, oat consumption can increase satiety and reduce energy intake, and can subsequently help promote the maintenance of a healthy weight by providing health-promoting dietary fibers [12,13,14].

Among the different types of dietary fiber, a relationship between the soluble fiber β-glucan and decreases in total- and LDL-cholesterol, risk factors for CVD, is well established [15,16,17]. For example, an FDA Health Claim for β-glucan, stating that 3 g of soluble fiber per day from oat foods in a diet low in saturated fat and cholesterol may reduce the risk of heart disease, has been allowed [18]. Notably, oats contain more soluble fiber than other grains, primarily in the form of β-glucan. A recent study showed that an oat beverage providing 3 g β-glucan daily could lower age-dependent systemic chronic inflammation and CVD risk to promote healthy aging in subjects with borderline high cholesterol [19,20]. Therefore, oats can have a beneficial effect on the inflammatory system and the aging trajectory in a subset of subjects presenting elevated risk factors. Many early studies on oat fiber and CVD also have reported reductions in BP, which was often attributed to β-glucan [11]. However, oats contain many other components that have been shown to beneficially impact metabolism, including phenolics (e.g., avenanthramides, flavonoids, tocols), a novel protein (avenalin), the anti-stress γ-aminobutyric acid (GABA), and minerals (potassium, magnesium) [7,17,21,22,23]. Therefore, the health benefits, although often attributed to β-glucan alone, may involve other components in oat products. The mechanisms underlying the attenuation of BP with oat interventions or the oat bioactives are also unclear [7,11]. This paper had two objectives: (1) to comprehensively review the clinical literature on oat consumption and BP management, and (2) to gain insight into oat bioactives with potential impact on BP. Further, this narrative review provides up-to-date information on oat bioactives present in different types of oat products and the potential mechanisms by which these oat components may function in healthy BP management.

## 2. Methods

### 2.1. Data Sources and Searches

Literature databases PubMed (Medline), Cochrane Central, and Google Scholar were searched in May 2022 using terms related to oats and BP. The search strategy is provided in Supplementary Table S1. The PubMed literature search was restricted to studies published in English or undetermined, with studies not in English excluded. For the Google Scholar search, only the first 400 titles were reviewed. No other limitations were placed on the literature search. Initially, these databases were systematically searched to identify systematic review and meta-analysis publications on oats and BP outcomes in humans. These reviews were perused for additional studies and included works by Llanaj et al. [24], Khan et al. [25], Kelly et al. [26], Hartley et al. [27], and Evans et al. [8]. References in recent comprehensive narrative reviews, including to Bouchard et al. [7], Alexandre and Miguel [11], and Singh and Belkheir [21], were also perused for relevant human studies. After the initial search, a systematic search for human clinical data on oats and BP outcomes was conducted.

### 2.2. Study Selection

The selection criteria of studies from the systematic literature search for clinical evidence included those that: (1) were human intervention trials, (2) were published in English, (3) provided information on a relevant clinical BP outcome (e.g., SBP, DBP, mean arterial pressure (MAP)), and (4) included interventions with an oat-based component alone. Studies that combined an oat intervention with dietary changes (e.g., low-fat, low-calorie, etc.) were included, but those that combined other foods or bioactives (e.g., wheat and oat, non-oat-based phenolic extracts) were excluded. Observational human studies were included for discussion only when clinical studies were unavailable.

The review of oat bioactives with putative effects on BP involved an extensive series of broader literature searches, including clinical evidence as well as animal and in vitro data. In addition, searches on relevant bioactives and phytochemicals from other sources (e.g., barley, fungi, etc.) and BP outcomes, as well as specific oat-based foods (e.g., sprouts), were also conducted. The most representative and recent publications were included in this discussion.

## 3. Results and Discussion

### 3.1. Reviews on Oats and Blood Pressure

The search for reviews identified four meta-analyses, of which three included subgroup assessments of oats on BP (Table 1). Overall, subgroup analysis for β-glucan sources showed reductions in SBP and DBP [8,25]. In a meta-analysis of normotensive adults, sensitivity analysis suggested that neither age nor body mass index (BMI) had a marked impact on the effect of fiber interventions and BP [8]. A separate meta-analysis including adults with HTN found no effects from fiber amounts, study design, and type of administration; although higher reductions were seen in interventions lasting more than 7 weeks, the reductions in BP were also influenced by the HTN status in subjects [25]. A recent meta-analysis of oat products, oat β-glucan-rich extracts, avenanthramides, and SBP and DBP outcomes, suggests that the effect of oats on BP is affected by the type of control or comparator [24]. Analysis comparing oat supplementation against the same/similar intervention without oats revealed no significant effect on either SBP or DBP. When comparing oat supplementation combined with dietary restriction (e.g., low-fat, hypocaloric) against dietary restriction alone, SBP was significantly different but increased, and DBP was decreased but did not reach significance. Comparing oats against heterogeneous controls (e.g., wheat, fiber, rice, eggs, etc.), the authors reported significant differences but not decreases for both SBP and DBP. More recently, Reynolds et al. [28] published a systematic review with meta-analysis that included a subgroup assessment of fiber and BP outcomes. The systematic review included nine studies on fiber and BP, of which four used an oat intervention [29,30,31]. Meta-analysis was conducted with eight of these studies, which included three interventions of oat and its effects on BP, and showed a beneficial effect of fiber on both SBP and DBP. 

Two other comprehensive reports that are worth noting were also identified. Thies et al. [32] included a listing of studies conducted on oats published through November 2012 that provided a measurement of BP [32]. These authors reported few studies with a significant effect on BP but noted that none of the studies was adequately powered to evaluate oats or oat bran on BP outcomes. Alexandre and Miguel [11] published a comprehensive review on fiber and BP and found several studies showing a beneficial effect utilizing oat-based interventions [11]. Relevant primary studies from these reports are provided in Table 2.

Taken together, these data support that responses are more robust in HTN than in normotensive subjects and that the amount of fiber/intervention product is essential. However, although the oat findings were attributed to β-glucan, the interventions utilized in the meta-analyses included studies primarily using oatmeal/porridge, oat cereal, and foods made with oat bran, not isolated β-glucan fiber. Therefore, although some conclusions were attributed to β-glucan effects, the other components in oats may also be contributing to the effect of lowering BP. Moreover, in some cases, these components were present in the comparators, which would confound the comparisons.

### 3.2. Human Clinical Studies on Oats and Blood Pressure

In addition to the 13 studies included in the previously discussed reviews, our search identified five other studies published within the past 10 years that have not been discussed in the published systematic reviews and meta-analyses. None of the five studies explicitly stated that the effect on BP was the primary outcome or that the study was powered to detect differences in BP, except for a 2021 study by Xue et al. [31]. This study assessed the effect of dietary fiber supplementation on gut microbiota in people with HTN (N = 50) using 30 g/d oat bran (8.9 g dietary fiber) with dietary guidelines for the intervention arm, compared to dietary guidance alone [31]. Significant decreases in multiple SBP and DBP measurements were seen in the oat compared to the diet-alone group after 3 months (Table 2). Notably, 31.8% of the subjects consuming oat bran decreased or discontinued medications for HTN and none increased medications, whereas ~9% in the diet alone group had anti-HTN medications increased during the study (*p* = 0.021). No significant differences were seen in the α-diversity of gut microbiota; however, at the end of the study, β-diversity was significantly different between the two groups (*p* = 0.019) [31]. Specifically, *Bifidobacterium* and *Spirillum* increased significantly in the oat group while decreasing in the diet-alone group. The authors speculate this could lead to differences in short-chain-fatty acid (SCFA) production but did not analyze SCFAs in the study. 

As noted in the reviewed systematic reviews and meta-analyses, effects are more often seen in people with elevated BP at baseline. Unsurprisingly, the four remaining studies that did not primarily investigate BP reported no significant changes in SBP or DBP. A 2019 pragmatic 6-week RCT comparing a low-energy diet (LED) with and without 40 g/d of oat bran in 154 people with metabolic syndrome found that both groups lost similar weight and showed similar decreases in SBP and DBP [45]. AlFaris and Ba-Jaber [43] reported on a 3-month RCT in 78 women with type 2 diabetes that compared six protocols combining LED and nutritional education with and without oat bran or oat bran and olive oil. A complication with this study is that the LED and counseling alone led to a significant difference; therefore, the effect of oat bran alone was confounding. Cicero et al. [44] published on 83 dyslipidemic people who were part of the Beta-glucan Effects on Lipid Profile, Glycemia and inTestinal Health (BELT) study. In this randomized 8-week intervention crossover study, subjects who were adherent to a Mediterranean diet consumed either 3 g/d oat β-glucan or an oat-based isocaloric placebo without β-glucan, matched for macronutrients. Pre-diet, the subjects were not generally hypertensive (SBP/DBP = 128/81 mmHg), and neither the placebo nor β-glucan supplementation affected BP. Finally, one study in normotensive, hypercholesterolemic people (*n* = 191) with a 1-g/d β-glucan product compared to rice reported no significant effect on SBP or DBP after 4 weeks [20]. 

Some reports that are not RCTs or full publications are worth noting. First, Damsgaard et al. [46] conducted a cross-sectional analysis of whole-grain consumption and cardiometabolic parameters in 713 Danish 8–11-year-olds. These authors reported no significant associations between whole grain and SBP or DBP when grain sources were combined; however, when assessed by source, whole grain oat was significantly associated with lower SBP (*p* = 0.02), whereas no significant difference was found for whole grain wheat or rye [46]. This study is the only report on BP, oats, and children found in this search and suggests the findings in adults may be relevant for younger populations, although more direct evidence is needed. Spencer et al. [47] published an abstract of a 3-arm double-blind, placebo-controlled 4-week crossover study in 28 people comparing (1) oatmeal/oatcake delivering 68.1 mg of oat phenolics, (2) oat bran concentrate with rice porridge/wheat cracker delivering 38.9 mg of phenolics, and (3) rice porridge/wheat cracker delivering only 13.8 g phenolic acids. In this study, significant improvements in SBP (−1.16 mmHg; *p* < 0.05) nighttime SBP (−5.1 mmHg; *p* < 0.01), and nighttime DBP (−2.3 mmHg; *p* < 0.05) were observed with the high phenolic oat intervention [47]. The authors also reported a non-significant improvement in microvascular function, as assessed by flow-mediated dilation (FMD; +1.09% ± 0.41%), and suggested the phenolic components in oats may, in part, play a role in factors such as BP modulation. This study, although published as an abstract only, is notable because studies have primarily used oats (e.g., whole oats, oatmeal, oat cereal), or oat bran, with only a few using concentrated oat β-glucan (Table 2). However, as shown in this study, other components in oat may contribute to the BP modulation effects of oat foods. 

Overall, decreases in BP are seen in subjects with elevated BP and in studies that are appropriately powered to detect differences. Most recently, when assessing HTN medications, data suggests interventions with oat products may help reduce the use of medications. However, data in normotensive subjects do not show consistent effects. Furthermore, although early studies implicated β-glucan as the active component, most interventions have been conducted with oat bran or whole oats. Therefore, oats contain other components that may be important for the effect on BP.

### 3.3. Oat Composition and Bioactives

Oat is unlike many other cereal grains based on its composition; it is primarily consumed as a whole grain, although oat bran is often used in many food applications [7]. Several oat varieties are grown across the globe and include husked oats (*Avena sativa* L.), large naked oats (*Avena nuda* I.), small naked oats (*Avena nudibrevis*), wild red oats (*Avena steriles*), and wild oats (*Avena fatua*) among others [7,21]. The composition of the oat is highly influenced by the oat genotype, growing conditions, and environment [7]. The oat grain from *Avena sativa* L. is the most common source for human consumption. The raw grain is composed of the groat, which contains the bran, endosperm, and germ, surrounded by the hull. The hull is removed for human consumption, although used for other industrial purposes, and when whole grain oat is consumed, it is in the form of the groat. It should be noted that, in addition to the hull, many other parts of the oat plant have been used for traditional medical applications and animal feed, including roots, straw, dried or fresh leaves, flowers, and stems [21]. Discussion of these components is beyond the scope of this review, although they may contain bioactives similar to those of some of the oat preparations used in human foods. The high nutritional value of oats has increased the interest in various novel oat products, such as germinated oats or oat sprouts [23,48,49]. During germination, the oat seed absorbs moisture, and as the seed softens, enzymes are activated, leading to changes in, or release of, nutrients that increase digestibility and bioavailability [23]. Along with the beneficial changes in the nutrition profile, sprouted oats also have improved palatability and enhanced flavor [23].

Oats are high in protein compared to other grains, which is found primarily in the bran with some in the germ [7]. The protein quality is also greater compared to that of many other plant sources, with higher lysine and lower proline and glutamine than other common grains, making it nearly the same quality as that of eggs, soy, milk, and meat proteins [7,21,23]. Oat groat is also composed of around 5–12% fats, primarily palmitic (16:0) and the unsaturated fatty acids, and oleic (18:1) and linoleic (18:2) acids [23]. Much attention has focused on the carbohydrate content of oats, primarily fiber. Compared to other major grains in human food consumption, oat is among the highest in fiber, delivering ~10.3 g of dietary fiber per 100 g of oats [7,21], of which 3–8 g is β-glucan [23]. Oats are also a source of tocols (tocopherols and tocotrienols), supporting vitamin E nutriture [21,50]. The amount of tocols in oats ranges from 0.5 to 3.61 mg/100g, with the most abundant tocopherol being α-tocopherol [50]. Oats provide vitamin A, β-carotene, vitamin B1, and vitamin B6, and the minerals potassium (355 mg/100 g seeds) and phosphorus (342 mg/100 g seeds), as well as calcium, magnesium, iron, zinc, copper, and manganese [21].

Oats also contain an array of phenolics, also called phytochemicals or phytonutrients, which have gained interest due to their potential impacts on human health. Notably, oat is the only food that contains avenanthramides, a group of phenolic alkaloids [22,23,51]. Soycan et al. [52] assessed the polyphenolics and avenanthramides in a range of commercial oat products available to consumers, including oat bran concentrate, flaked oats, and rolled oats [52]. Oat bran concentrate had the highest amounts of phenolic acids and avenanthramides, although all oat foods had relatively high amounts of these bioactives, delivering 15.79–25.05 mg total phenolic acids and 1.1–2.0 mg avenanthramides in a 40 g portion of oat product [52]. 

In an extensive systematic review, Raguindin et al. [50] summarized 72 studies on oat phytochemicals and metabolism, finding that most compositional reports were published in the 2010s. These authors also noted that different protocols, use of solvents, preparation (e.g., heating, microwave), and source (e.g., growing conditions, genetics) impact the amounts of phytochemicals found in various studies. However, the authors found some consistency and cataloged the range of phytochemicals in various oat plant parts, including seeds, bran, flour, and whole grout. Overall, 16 different flavonoids, 9 phenolic acids, 16 phytosterols, and 24 different alkaloids have been identified in these oat foods [50]. The main polyphenol compounds found in oats include protocatechuic, syringic, vanillic, *p*-hydroxybenzoic, gallic, *p*-coumaric, o-coumaric, and caffeic acids. The most common phenolic acid in oats is ferulic acid (~150 mg/100 g oats) [23,50], and the most abundant flavonoid is quercetin (up to 8.9 mg/200 g husked oat) [50], both of which are found in many other grains. As mentioned above, avenanthramides are unique to oats, and more than 20 forms are found in oats, with the most abundant esters being 5-hydroxyanthranilic acid and *p*-coumaric (AVA-A), ferulic (AVA-B), and caffeic (AVA-C) acids [22,50,53]. These phenolics have been suggested to have beneficial effects on BP (discussed below). In addition, oat is the only cereal with saponins, which are also found in some other non-grain plants and have emerging health benefits [50].

### 3.4. Oat Sprouts as a Source of Bioactives

Oat sprouts have been shown to maintain the high-quality protein characteristics and similar fiber content of whole oats [54]. Proteins are one of the major fractions of grains and these are strongly affected by germination, whereby storage proteins are broken down to provide small peptides and amino acids to the growing embryo. Oat germination was shown to increase total protein and both essential and non-essential amino acid contents, although these are dependent on germination conditions (e.g., temperature and duration) [55,56]. Germination also increased protein digestibility [57]. This is important in light of the low protein digestibility of plant proteins in general [58]. Although fiber content is also consistent between oat grain and sprouted oat, germination leads to significant hydrolysis of β-glucan, possibly due to the increased activity of endogenous β-glucanases, resulting in a decrease of around 40% [56].

Sprouted oats contain much higher levels of important minerals, including calcium, iron, zinc, and magnesium, and interestingly, up to 30-fold higher levels of GABA [54,59,60]. Sprouted oats show an increase in free phenolics such as avenanthramides, with between 3- and 5-fold higher phenolic content, which could be due to de novo synthesis or release of the compounds from the cell wall and fiber components [54,61,62]. Due to the beneficial nutritional profile and positive sensory characteristics of sprouted oats, germination of oat products is being explored as a base for innovative functional foods, including gluten-free fermented functional beverages [54].

### 3.5. Antihypertensive Effects of Oats: Putative Mechanisms

**Figure 1:**
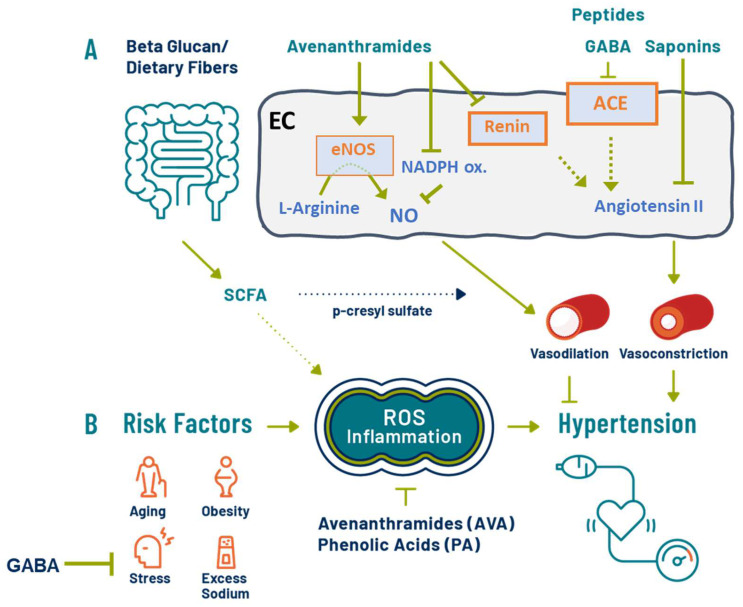
Mechanism of action of the anti-hypertensive effects of oat bioactives. (**A**) Gut microbiome-mediated metabolism of oat dietary fibers can increase circulating short-chain fatty acids (SCFA) involved in the reduction of hypertension, in part via reduction of *p*-cresyl sulfate, associated with vascular function (vasodilation) and cellular oxidative damage. In the endothelial cells (EC), avenanthramides (AVA) and phenolic acids (PA) induce vasodilation and enhance vascular function by endothelial nitric oxide (NO) synthase (eNOS) and by inhibiting NADPH oxidases’ regulation of NO degradation. Furthermore, angiotensin II-mediated vasoconstriction, endothelial dysfunction, and hypertension can be directly inhibited by oat saponins or indirectly by AVA inhibition of renin and GABA inhibition of angiotensin-converting enzyme (ACE) upstream of angiotensin II. GABA, AVA, and oat saponin can help improve endothelial cell function. (**B**) Risk factors can elicit a pathological rise of systemic inflammation and elevated intracellular levels of reactive oxygen species (ROS) that are critically implicated in the etiology of hypertension. Oats’ PA and AVA are anti-inflammatory and antioxidant bioactives that could contribute to the maintenance of a healthy BP.

BP control involves a complex interplay among renal function, vascular health, and neural regulatory pathways, arising from genetic factors and diet and lifestyle effects. Some data suggest that early-life factors, such as maternal nutrition and early-life oxidative stress, can lead to HTN in adulthood [63]. Pathophysiological conditions or poor lifestyle choices can elicit oxidative stress and increase levels of reactive oxygen species (ROS) that are a major contributor to vascular dysfunction and remodeling, leading to HTN (Figure 1). In particular, ROS decrease nitric oxide (NO) synthesis, and HTN is associated with a reduction in antioxidant capacity and bioavailability. Treatments to manage high BP are equally complex and often involve multiple, personalized modalities. Diet and lifestyle are cornerstones of public health recommendations for prevention and components of therapeutic programs to manage HTN. For example, initial guidelines recommend diet and lifestyle alterations as the first step in mitigating elevated BP and Stage 1 HTN, followed by the inclusion of pharmacological and more targeted approaches for non-responsive cases and above Stage 1 HTN [2]. Traditional lifestyle changes include weight management, smoking cessation, low-sodium diets, and decreasing alcohol and caffeine consumption [6]. Plant-based diets are also associated with lower risk of HTN [64]. However, it is becoming increasingly clear that functional ingredients in foods, such as dietary fibers, phenolic acids, functional peptides, and amino acids, can impact BP through numerous mechanisms (Figure 1). For example, the use of dietary antioxidants such as vitamins C and E, and polyphenols, has gained considerable interest as protecting agents against vascular endothelial dysfunction via attenuation of ROS production. 

### 3.6. Dietary Fiber and SCFA

Diets high in fiber, particularly soluble fibers (e.g., β-glucan), are recommended to decrease elevated BP [8,11,25,27,28,65,66], and some evidence suggests they function by decreasing glucose uptake and therefore decreasing insulin release [67]. Emerging evidence suggests a healthy microbiome is also important in BP management [7,15,68,69]. For example, SCFAs produced by the microbiota have been shown to have vasodilating effects and anti-inflammatory properties, which can attenuate hypertensive tissue damage [7]. In a clinical pilot study, increases in fecal SCFA decreases serum *p*-cresyl sulfate, and improvements in endothelial function via flow-mediated dilation (FMD) have been observed after consumption of a pasta enriched with barley β-glucans [70,71]. However, in another study with 210 mildly hypercholesterolemic Chinese adults, 80 g oats delivering 3 g β-glucans and 56.8 g polyphenols did not significantly change plasma SCFA but did result in putative beneficial changes in gut microbiota after 45 days [72]. Further, in vitro and animal studies have found SCFA can stimulate vascular and renal G-protein–coupled receptors 41 and 43 (GPR-41/43), decrease systemic inflammation and atherosclerotic lesions, and enhance protective responses to endothelial production of ROS induced by angiotensin II [7,68,69,73,74]. 

Although β-glucan has received much of the focus due to its established role in cholesterol attenuation, in vitro evidence suggests other components of oats may impact the microbiota and production of SCFA. For example, Kristek et al. [75] conducted in vitro fermentation studies on oat bran, isolated oat β-glucans, and polyphenols and found oat bran led to more production of SCFAs than β-glucans, and polyphenols alone did not result in appreciable levels. In particular, the oat bran, which contains both β-glucans and polyphenols, increased the relative abundance of *Bifidobacterium adolescentis*, which can synthesize and secrete GABA [75]. Additionally, in vitro fermentation studies indicate that oat bran β-gluco-oligosaccharides enhanced the growth of three lactic acid bacteria (i.e., *Lactobacillus rhamnosus*, *Lactobacillus plantarum*, and *Lactococcus lactis*) as well as the production of lactic acid bacteria fermentation end-products [76]. More importantly, the abundance of certain *Lactobacillus* species is negatively associated with BP [77,78,79].

### 3.7. Oat Phenolics and Avenanthramides

In epidemiologic and clinical studies, categories of phenolic compounds are increasingly promoted to help manage a healthy BP due to the positive correlations with a plant-based diet rich in whole grains, fruits, and vegetables. For example, Kay et al. [80] classified phenolics by flavonoid category and found a significant reduction in SBP (mean difference, −1.46 mmHg; 95% CI, −2.38, −0.53) and DBP (mean difference, −1.25 mmHg; 95%CI, −1.82, −0.67) when data across all flavonoids were pooled. Subgroup analyses indicate that this positive response was mainly driven by epicatechin, quercetin, and procyanidins. However, in a meta-analysis of phenolics and BP, Godos et al. [81] reported that anthocyanins were associated with the reduced risk observed by the flavonoid class of polyphenols, whereas results with the flavanol subclass (e.g., quercetin) were null. Therefore, the role of oats’ phenolic acids in managing BP is still emerging.

Avenanthramides have established physiological properties, including anti-inflammatory, antioxidant, and anti-thrombotic benefits, with reportedly higher antioxidant activity compared to that of other phenolic compounds in vitro [22,23]. Cell culture studies have found that avenanthramides can increase NO levels and endothelial NO synthase expression in vascular smooth muscle cells and endothelial cells, thus potentially affecting NO-dependent vasodilation [17,62]. In vitro data suggest the effect on NO may occur via reducing cellular superoxide levels, or acting as NADPH oxidase inhibitors, thereby reducing NO degradation [82]. Avenanthramides have also been shown to inhibit the adhesion of monocytes to vascular endothelial cells and release inflammatory activators from macrophages, and exert anti-proliferative and pro-apoptotic activities in transformed cells [51,83]. Increasingly, inflammation is being linked to HTN and, particularly hypertensive tissue damage [84]. Further, monocytes, macrophages, and dendritic cells of the immune system can promote elevations in BP [84]. Therefore, the potential beneficial impact of oat polyphenols on inflammation and immune function could also contribute to the attenuation of elevated BP.

Studies in humans with oat products generally support these findings, with oat interventions suggesting beneficial effects on FMD and/or promoting healthy NO levels, although findings have not always reached significance [47,70,85,86,87,88]. The amount of oat phenolics consumed in these past studies, however, has not been reported, with exception of the report from Spencer et al. [47] (discussed above). This study assessed preparations delivering 13.8 mg, 38.9 mg, and 68.1 mg of oat phenolics and found a beneficial effect on BP with the highest amount of oat phenolics [47].

Bioavailability studies have shown that avenanthramides and numerous phenolics are present in human blood and excreted in urine after consumption of oat bran [89,90,91,92]. The mean intake of oat avenanthramides is estimated at between 0.3 mg/d and 2.1 mg/d [93]. Germination of oat seed (i.e., oat sprouts) is known to enhance levels of free phenolics, particularly avenanthramides, by up to 30-fold [54,60]. Therefore, oat sprouts appear to have higher levels of beneficial phenolics such as avenanthramides. Animal studies support the role of avenanthramides from oat sprouts in decreasing inflammation in a colorectal cancer model [61]. Furthermore, avenanthramides can be biotransformed into dihydro-avenanthramides, a potent antioxidant polyphenol, especially in individuals with a significant enrichment of gut bacteria *Faecalibacterium prausnitzii* [90,94]. These findings may explain some of the individualized physiological responses to oats or oat sprout consumption. 

### 3.8. Oat Bioactive Peptides and γ-Aminobutyric Acids (GABA)

Oats are the only cereals to contain the globulin or legume-like protein avenalin, as well as gluten, zein, and avenin proteins [21]. Aside from providing protein nutriture, emerging in vitro and in silico data indicate oats contain bioactive peptides. For example, in vitro and animal studies suggest oat protein-derived peptides have broad antioxidant activity and can inhibit cyclooxygenase-1, α-amylase, and DPP-IV activities, suggesting beneficial effects for supporting cardiometabolic health [7,95,96,97,98,99,100]. Specific to BP, an oat globulin peptide (peptide SSYYPEK, 890.4 Da) prepared by hydrolysis has shown the ability to inhibit angiotensin-1-converting enzyme (ACE) activity and suppress renin and intracellular endothelin-1 in spontaneously hypertensive rats at 100 to 150 mg/kg body weight [101]. Peptides of different oat protein isolates were investigated by in silico hydrolysis and chemical synthesis methods with several peptides and found to inhibit the ACE enzyme (by 86.5–96.5%) and renin (40.5–70.9%) in vitro at 1 mg/mL [100]. Moreover, strong ACE-inhibitory activity has been found in oat bioactive peptides (<3 kDa) produced by simulated gastrointestinal digestion, with the half-maximal inhibitory concentration (IC50) for different hydrolysates of 35 and 85 μg/mL [102]. The ACE is an important target for many BP interventions as it regulates the renin–angiotensin–aldosterone system, which is the primary metabolic pathway controlling arteriolar vasoconstriction and intravascular fluid volume and, thus, BP [100]. 

Much interest has been generated around GABA, which is a non-protein amino acid with numerous beneficial activities, including reducing stress and enhancing sleep in human trials [103]. GABA shows great promise as an intervention to help manage healthy BP and has a positive safety profile [103,104]. Although GABA is an important neurotransmitter, studies have shown that dietary GABA does not appreciably transit the blood–brain barrier; therefore, the mechanism(s) supporting these clinical findings do not relate to the effect on brain chemistry [104]. In vitro studies support the effect of GABA as an ACE inhibitor at IC50 values up to 0.70 ± 0.07 mg/mL, and studies in spontaneously hypertensive rats at 0.3–300 mg/kg (intraduodenal) GABA have shown dose-dependent decreases in BP [104]. In humans, clinical studies on fermented foods, such as fermented milk and soy products, delivering between 0.25 mg/d and 18,000 mg/d have been published and shown significant decreases in BP (<10% change), with BP returning to baseline levels when the food intervention was discontinued [103]. As noted above, increased levels of GABA are found in oats after fermentation or germination [49,54,60,105,106]. Sprouted oat powder has been shown to contain ~55 mg GABA/100 g [54], which is within the range seen as active in lowering BP in clinical studies [103]. 

### 3.9. Other Oat Components: Saponin

Saponins are steroidal components that, similar to phenolics, are produced in the plant as defensive compounds [67]. Consistent with their role as a protectant, saponins have potent in vitro antioxidant activity, and saponins from oat seed roots have antifungal properties [67]. The types of saponins in oat are just being elucidated, with at least two unique structures (steroidal saponins, avenacoside A, 1, and avenacoside B, 2) and up to 11 others present in different oat products [107,108,109]. Limited information is available on the bioactivity of oat-based saponins, although some in vitro data has shown the ability of oat saponins to inhibit the growth of human colon cancer cells [109]. More information is available on saponins from other plant sources, and emerging data suggest they may function as anti-obesity agents via inhibition of lipase, modulation of adipogenesis, and/or influencing energy intake [110]. More directly, saponins have been found to inhibit angiotensin II activity in vitro and in spontaneous hypertensive rat models [111,112]. Therefore, oat saponins may also support healthy BP, although more data are needed to understand levels of saponins in different oat products and their efficacy in humans.

## 4. Summary

Overall, available clinical data support the beneficial effect of oats on BP in adult subjects with elevated BP and in studies that are appropriately powered to detect differences. Moreover, the data also suggest interventions with oat products may help reduce the use of anti-HTN medications. A consistent effect of oats on BP lowering was not found in normotensive subjects. No studies on the effect of oats on BP in children were identified.

The effect of oats on BP may not be surprising since oats are a source of high-quality, plant-based protein and some data suggest plant-based diets are beneficial for healthy BP. Much attention has also been focused on diets high in fiber and BP management, and oats are also high in fiber, particularly the soluble fiber, β-glucan. Given the established role of β-glucan in attenuating elevated blood cholesterol, which helps lower the risk of CVD, it is possible that β-glucan could also be important for lowering BP. Notably, the vast majority of studies were conducted with whole oats or oat bran-based foods, which could deliver not only β-glucan but also these other oat bioactives. Indeed, oats are a unique source of avenanthramides, phenolic amides containing anthranilic acid and hydroxycinnamic acid moieties, which have shown antioxidant, anti-inflammatory, and antiproliferative effects which have direct and indirect effects on BP. Therefore, the effect of oats on BP may be due to components beyond fiber and, specifically, β-glucan.

In particular, oats are a dietary source of GABA, which has known effects on BP. Although not present at a high level in whole oats, GABA levels are increased up to 30 times in germinated or sprouted oats. Therefore, further research on oats, specifically sprouted oats, and BP is warranted. The ability of oat-based peptides to inhibit ACE-I is also intriguing, although these findings are from in vitro studies and require follow-up in clinical trials. 

A strength of this report is the focus on oats and BP and the inclusion of human data along with a thorough assessment of oat composition and putative bioactive mechanisms. Although this was a comprehensive review with a systematic approach to the search and selection of the evidence, a limitation is that it was not a systematic review of the evidence given the quality of the individual studies was not assessed. This report has identified the need for future clinical studies to provide clear information on the composition of the oat foods used in studies beyond the amount of fiber and β-glucan. In addition, many of the studies on normotensive subjects included BP assessment as a secondary outcome, and therefore, may have been underpowered to see an effect in this population. Thus, future studies in adults with modestly elevated BP that are appropriately powered should be conducted to understand the effect of oats on the general population. Overall, oats, particularly foods with sprouted oats, in which phenolics such as avenanthramide and GABA are enhanced, hold promise for a beneficial impact on BP management and overall health.

## Figures and Tables

**Table 1:** Meta-analyses reports on oats and blood pressure.

Citation	Purpose/Description	Conclusions on Oats
Evans et al. [8]	Assessed the effect of different types of fibers on BP. RCTs of at least 6 wks duration, testing a fiber isolate or fiber-rich diet against a control or placebo. Included 5 and 4 studies on oat β-glucan rich interventions for SBP and DBP analyses, respectively, published between 1 January 1990 and 1 December 2013.	SBP: Combined fiber interventions resulted in a significant (*p* = 0.023) decrease of −0.92 (95% CI: −2.48, 0.63). The subgroup analysis of oats or β-glucan-rich intervention showed a decrease of −2.69 (95% CI: −4.6, −0.73). DBP: Combined fiber interventions resulted in a significant (*p* = 0.001) decrease of −0.71 (95% CI: −1.90, 0.47). The subgroup analysis of oats or β-glucan-rich intervention showed a decrease of −1.45 (95% CI: −2.68, −0.22). Higher consumption of β-glucan fiber is associated with both lower SBP and DBP, with a median difference of 4 g. Data suggests much of the effect of fiber on BP may be driven by β-glucan-rich fiber sources.
Llanaj et al. [24]	Assess the effects of oat supplementation interventions on CVD risk factors. RCTs that included oat, oat β-glucan-rich extracts, and avenanthramides on CVD risk markers (e.g., blood lipids, glucose, BP). Included 5 studies comparing oats vs. diet or no-oats control, 5 studies comparing dietary restriction with and without oats, and 7 studies of oats vs. a heterogeneous control, published from database inception until 15 May 2020.	SBP: Decreased non-significantly (WMD, −0.56 mmHg; 95% CI −1.68 to 0.56; *p* = 0.20; *I*^2^ = 33.8) when oats compared to similar diet or product intervention, and a slight significant increase for oats with restrictive diet compared to diet alone (WMD, 0.170 mmHg, 95% CI −2.168 to 2.508; *p* < 0.001; *I*^2^ = 88.3) or comparison to heterogeneous (e.g., wheat, fiber, rice, eggs, etc.) interventions (WMD, 0.547 mmHg; 95% CI −0.564 to 1.657, *p* < 0.001; I^2^ = 85.3). DBP: Decreased non-significantly (WMD, −0.69 mmHg, 95% CI −1.59 to 0.22; *p* = 0.14; *I*^2^ = 42.8) when oats compared to similar diet or product intervention and for oats with restrictive diet compared to diet alone (WMD, −1.154 mmHg, 95% CI −2.030 to −0.0278; *p* = 0.060; *I*^2^ = 55.9), and significant slight increase for comparison to heterogeneous (e.g., wheat, fiber, rice, eggs, etc.) interventions (WMD, 0.357 mmHg; 95%CI −1.210 to 1.925; *p* < 0.001; *I*^2^ = 96.8). The effect of oat supplementation on BP was inconsistent, similar to that observed for glucose, and further high-quality trials are warranted.
Khan et al. [25]	Investigate the effects of viscous soluble fiber supplementation on BP and quantify the effects of individual fibers. Studies that assessed interventions of viscous soluble fiber supplements or diets enriched with soluble fiber on BP outcomes with durations of at least 4 wks. Included 8 studies on oat-soluble fiber published through 10 June 2017.	SBP: Assessment of the combined viscous fibers results in a significant reduction (MD, −1.59; 95% CI, −2.72, −0.46; *p* < 0.01), with subgroup analysis for β-glucan showing a similar decrease, although not significant (−1.50; 95% CI, −3.16, 0.16; *p* = 0.08). DBP: Assessment of the combined viscous fibers results in a modest reduction (MD, −0.39; 95% CI, −0.76, −0.0; =0.05), with subgroup analysis for β-glucan showing a greater decrease (−1.02; 95% CI, −2.06, 0.01; *p* = 0.05).
Reynolds et al. [28]	Examine the evidence for diets high in fiber on the management of CVD or HTN. Controlled trials of at least 6 wks duration of increasing fiber intakes in subjects with CVD or HTN and reporting on cardiometabolic risk factors. Nine studies assessed fiber in subjects with HTN for the systematic review and eight studies were used in the meta-analysis. Four studies used oats as the fiber source.	SBP: Decreased for fiber (MD, −4.3 mmHg, 95% CI −5.8 to −2.8) for combined fiber types, with studies assessing oats contributing ~34.3% of weighted contribution. DBP: Decreased for fiber (MD, −3.1 mmHg, 95% CI −4.4 to −1.7) for combined fiber types, with studies assessing oats contributing ~34.2% of weighted contribution. The effect of oat supplementation on BP was not assessed separately.

Abbreviations: BP, blood pressure; CI, confidence interval; CVD, cardiovascular risk; DBP, diastolic blood pressure; HTN, hypertension; MD, mean difference; RCT, randomized clinical trials; SBP, systolic blood pressure; wk(s), week(s); WMD, weighted mean difference.

**Table 2:** Clinical studies published on oats with blood pressure outcomes.

Citation (Δ)	Location	Subject Characteristics *	Trial Design/ Duration §	Oat Intervention	Control/ Comparator	BP Change mmHg (95% CI) †
Saltzman et al. [14] (1, 2, 3)	USA	Generally healthy N = 43: 49% male, Age: 20–70 y BMI: 20–35 kg/m^2^ SBP: 118 (15) mmHg DBP: 70 (8) mmHg Stratified on age (18–30 y; 60–75 y), sex, and BMI	BP co-primary parallel 6 wk 2 arms	45 g/1000 kcal of rolled oats (~3.7 g/d fiber) total fiber = 16 g/d (7.2 g soluble fiber)	45 g/1000 kcal of wheat products total fiber = 12.5 g/d (3.5 g soluble fiber)	SBP: WMD, −5.00 (−9.25, −0.75) DBP: WMD, −1.00 (−8.68, 6.68) Oat: ‡ SBP: 120 (110–130) mmHg to 110 (100–120) mmHg (*p* < 0.05) DBP: 80 (70–90) mmHg to 70 (70–80) mmHg (*p* < 0.05) Wheat: ‡ SBP: 120 (115–130) mmHg to 110 (105–120) mmHg (*p* < 0.05) DBP: 80 (80–85) mmHg to 70 (70–80) mmHg (*p* < 0.05) Δwt = NR
Wolever et al. [20]	Canada	Hypercholesterolemic N = 191: 37.7% Male Age: 47.6 (11.4) y BMI: 27.9 (4.6) kg/m^2^ SBP: 121 (13) mmHg DBP: 76 (9) mmHg	BP secondary parallel randomized double-blind 4 wk 2 arm	1 g oat β-glucan (produced by treating an oat product enzymatically to reduce starch content without affecting MW of β-glucan)	Rice milk powder	Oat β-glucan: ‡ SBP: 120 (1) mmHg to 121 (1) mmHg (ns) DBP: 75 (1) mmHg to 76 (1) mmHg (ns) Control: ‡ SBP: 119 (2) mmHg to 120 (1) mmHg (ns) DBP: 76 (1) mmHg to 77 (1) mmHg (ns) Δwt = ns
Pins et al. [29] (2, 3, 4)	USA	HTN subjects N = 88: ~50% Male Age: 30–66 y BMI: 30.6–31.2 kg/m^2^ SBP: 139 (16) mmHg DBP: 87 (10) mmHg Stratified by baseline SBP and soluble fiber	BP Co-primary parallel randomized single-blind 12 wk 2 arms >6 wk follow-up	60 g/d oatmeal (5.61 g/d dietary fiber, 3.25 g/d soluble fiber, 3.25 g/d β-glucan) + 77 g/d oat squares (6.07 g/d dietary fiber, 2.98 g/d soluble fiber, 2.59 g/d β-glucan)	65 g/d hot wheat cereal (2.32 g/d dietary fiber, 0.6 g/d soluble fiber) + 81 g/d corn and rice RTE cereal (1.2 g/d dietary fiber, <0.5 g/d soluble fiber)	SBP: WMD, −6.00 (−11.80, −0.20) DBP: WMD −5.00 (−12.17, 2.17) Anti-HTN medication ≥50% decreased or discontinued by 73% vs. 42% (*p* < 0.05) in oat vs. control groups, respectively. During post-study follow-up, 67% vs. 33% (*p* < 0.05) resumed medications in oat vs. control groups, respectively. Changes in those without medication: ‡ ΔSBP: oat, −7 (8) mmHg; control −1 (9) mmHg; *p* < 0.05 ΔDBP: oat, −4 (5) mmHg; control, +1 (6); *p* = 0.18 Δwt: ns
He et al. [30] (1, 2, 3, 4)	USA	HTN subjects N = 110: 40% male, Age: 37–60 y BMI: 23–34 kg/m^2^ SBP: 116–140 mmHg DBP: 73–85 mmHg %HTN: ~15%	BP primary parallel double-blind 12 wk 2 arms	60 g/d OB in muffins and 84 g/d oatmeal cereal squares delivering 7.3 g/d β-glucan. Total fiber: ~15.9 g/d (7.3 g as β-glucan)	93 g refined wheat in muffins and 42 g cornflake cereal. Fiber: 2.7 g/d (no β-glucan)	SBP: WMD, −1.8 (−4.3, 0.8; *p* = 0.17) DBP: WMD −1.2 (−3.0, 0.5; *p* = 0.17) After adjustment of the mean difference (95% CI) for age, race, sex, baseline BP, fiber, and wt, a change in wt resulted in a reduced net change for SBP of −0.5 mmHg (−2.7, +1.7; *p* = 0.64) and DBP of −1.0 mmHg (−2.0, +0.6; *p* = 0.24) Within-group analysis suggested a significant decrease in the high-fiber group for SBP (−3.4 mmHg; 95%CI, −5.2, +1.7; *p* < 0.001) and DBP (−2.1; 95%CI, 3.1, +1.0; *p* < 0.001) for the high-fiber group, and a non-significant change for SBP (−1.4 mmHg; 95%CI, −3.0, +0.2; *p* = 0.08) and DBP (−1.1 mmHg; 95%CI, −2.2, +0.1; *p* = 0.07) for the low-fiber group Δwt (kg) = 0.1 kg on oat intervention, 0.7 kg on control, *p* = 0.07 for difference
Xue et al. [31] (4)	China	HTN subjects N = 50: 72.7% Males Age: 47 (13) y BMI: 24.9 (2.5) kg/m^2^ SBP: 140–159 mmHg DBP: 90–99 mmHg 31.8% on anti-HTN medications	BP co-primary randomized 3 mo 2 arms 1 wk run-in	Arm 1: 30 g/d OB DASH diet guidance	Arm 2: DASH diet guidance	Office BP measurements for oats: ‡ SBP: 138.0 (11.1) mmHg to 122.6 (8.8) mmHg DBP: 91.7 (11) mmHg to 81.5 (7.7) mmHg Office BP measurements for control: ‡ SBP: 137.2 (10.1) mmHg to 133.0 (7.4) mmHg DBP: 86.8 (9.9) mmHg to 87.4 (9.2) mmHg Between-group mean differences for SBP and DBP were ns at baseline and significant (*p* < 0.001, *p* = 0.00) at end of the study, respectively. No significant differences in 24 min SBP or 24 min DBP. Significant mean differences for 24 h max and 24 h average SBP and DBP. Anti-HTN medications: control, 9.1% increased, oat, 0% increased, 31.8% decreased or discontinued (*p* = 0.021) Δwt = NR
Charlton et al. [33] (1, 2)	AUS	Dyslipidemic subjects N = 90: 48% Male, Age: 49.75–52.43 y BMI: 27.74–26.74 kg/m^2^ SBP: 132.12–128.76 mmHg DBP: 78.21–76.39 mmHg	BP secondary parallel randomized single-blind 6 wk 3 arms	Arm 1: High oats (3.24 g/d β-glucan) Arm 2: Low oats (1.45 g/d β-glucan) RTE oat flakes, oat cereal bars, oat porridge LFaD	Arm 3: Control (0 g/d β-glucan) RTE cornflakes, puffed rice bars LFaD	SBP (combined): WMD, 0.30 (−8.19, 8.79) SBP Mean change (mmHg) over 6 wk: Arm 1, −5.6; Arm 2, −4.7, Arm 3, −8.9; ns DBP: NR Δwt (kg): =0.7 kg
Davy et al. [34] (1, 2, 3)	USA	HTN subjects N = 36: 100% Male, Age: 57–61 y BMI: 29–30 kg/m^2^ SBP:133.7–138.2 mmHg, DBP: 88.1–88.5 mmHg, Fiber: 17–23 g/d	BP primary parallel randomized 12 wk 2 arms	60 g oatmeal and 76 g OB RTE cold cereal Dietary fiber: 14 g/d β-glucan: 5.5 g/d	Wheat-based RTE cold cereal control, Dietary fiber: 14 g/d β-glucan: 0 g/d	SBP: WMD, −5.70 (−13.51, 2.11) DBP: WMD, −3.30 (−4.34, 2.32) No significant difference for SBP or DBP in daytime casual and supine arterial, 24-ambulatory, variability, nocturnal dip, load, or pulse rate. For SBP, no differences in nighttime or MAP, but a small, significant, increase in nighttime DBP and MAP in both placebo and β-glucan. Δwt (kg): 0.2
Liao et al. [35] (3)	Taiwan	Mild hypercholesterolemic N = 48 hypercholesterolemic and N = 34 normal: Age: 38–76 y BMI: 23.38–23.66 kg/m^2^ SBP: 116.95–126.23 mmHg DBP: 77.73–78.91 mmHg	BP secondary parallel randomized double-blind 10 wk 2 arms	100 g/d (366 kcal) Oat noodles (80% oat, 3.12 g/d β-glucan, 20% wheat flour)	100 g/d (190 kcal) Wheat noodles (100% wheat flour)	Oat noodles: ‡ SBP: 126.23 (3.81) to 112.38 (4.12) mmHg (*p* < 0.01) DBP: 78.91 (1.89) to 73.00 (2.31) mmHg (*p* < 0.05) Wheat noodles: ‡ SBP: 116.95 (2.42) to 119.50 (3.03) mmHg (ns) DBP: 77.73 (2.07) to 73.33 (1.76) mmHg (ns) Δwt: No change for both groups (range 58.66 kg to 58.11 kg)
Maki et al. [36] (1, 4 SR only)	USA	Elevated SBP and/or DBP N = 97: 55% Male Age: 56–62 y BMI: 32.2–32.6 kg/m^2^ SBP: 138.9, 139.9 mmHg DBP: 82.8, 83.9 mmHg Fiber < 20 g/d	BP primary parallel randomized double-blind 12 wk 2 arms	90 g/d OB cereal + 60 g/d oatmeal + 20 g/d powdered oat β-glucan Total dietary fiber: 17.3 g/d (7.7 g/d β-glucan)	90 g/d Low-fiber RTE (wheat) cereal + 65 g/d Low fiber hot cereal + 12 g/d placebo supplement Total dietary fiber: 1.9 g/d fiber (no β-glucan)	SBP: WMD, −3.80 (−8.78, 1.18) High-BMI (>31.5 kg/m^2^) subgroup analysis: significant between-group difference for ΔSBP (oat, −5.6 mmHg; control, +2.7 mmHg; *p* = 0.008) and ΔDBP (oat, −2.1 mmHg; control, +1.9 mmHg; *p* = 0.018) No between-group significant differences for SBP or DBP overall, or for the lower-BMI subgroup analyses Δwt: <1 kg for both groups, ns
Onning et al. [37] (2, 3)	Sweden	Dyslipidemic subjects N = 52: 100% males Age: BMI: kg/m^2^	Crossover, double-blind 5 wk	Oats delivering 3.8 g/d β-glucan	Rice	SBP: WMD, −1.60 (−5.50, 2.30) DBP: WMD −1.50 (−2.44, −0.56) Δwt = +1%, *p* < 0.001
Queenan et al. [38] (2, 3)	USA	Dyslipidemic subjects N = 75: 33% males Age = ~45 y BMI = NR SBP: 121 mmHg DBP: 67–69 mmHg	BP secondary parallel randomized double-blind 6 wk 2 arms	6.0 g/d concentrated oat β-glucan supplement (from 12 g OB concentrate)	6 g/d placebo (dextrose) supplement	SBP: WMD, 0.00 (−0.10, 0.10) DBP: WMD 0.00 (−0.12, 0.12) Oat β-glucan: ‡ SBP: 121 (2.2) mmHg to 119 (1.9) (ns) DBP: 69 (1.4) mmHg to 69 (1.3) mmHg (ns) Placebo: ‡ SBP: 121.6 (1.9) mmHg to 119 (2.0) mmHg (ns) DBP: 67.1 (1.5) mmHg to 69 (1.7) mmHg (ns) No significant difference between groups at end of the intervention Δwt: placebo, 1.4 (1.3) kg, oat, −0.7 (0.3), ns
Momenizadeh et al. [39] (3)	Iran	Hypercholesterolemia N = 60: 35% Males Age: 51.12 (9.31) y BMI: 28.94–28.99 kg/m^2^ SBP: 114.83–115.17 mmHg DBP: 76.33–77.00 mmHg	BP secondary parallel randomized 6 wk 2 arms LED 2 wk run-in	150 g/d OB bread (30 g/d β-glucan) + LED	150 g/d wheat bread (no β-glucan) + LED	oat: ‡ SBP: 114.83 (10.95) mmHg to 112.50 (12.16) mmHg (ns) DBP: 77.00 (9.15) mmHg to 76.33 (8.90) mmHg Control: ‡ SBP: 115. 17 (14.65) mmHg to 114.83 mmHg (13.55), ns DBP: 76.33 (10.74) mmHg to 75.33 (9.37) mmHg No significant difference in SBP or DBP between groups
Raimondi de Souza et al. [40] (3)	Brazil	Hypercholesterolemia N = 132: 33% Male Age: 40–70 y (~30% <60 y) BMI: 25–35 kg/m^2^ SBP: 120 mmHg DBP: 80 mmHg Fiber: 21.7–22.3 g/d	BP secondary randomized double-blind 90 d 2 arms	40 g OB (β-glucans) in fat-free powdered milk + nutrition counseling	Placebo: 40 g corn starch and rice flour + nutrition counseling	Oat β-glucan: SBP: 120 (110–130) mmHg to 110 (100–120) mmHg (*p* < 0.05) DBP: 80 (70–90) mmHg to 70 (70–80) mmHg (*p* < 0.05) Placebo: SBP: 120 (115–130) mmHg to 110 (105–120) mmHg (*p* < 0.05) DBP: 80 (80–85) mmHg to 70 (70–80) mmHg (np < 0.05) No significant difference in BP between groups. Δwt = oat, −3.5 kg (*p* < 0.05); placebo, −2.0 kg (*p* < 0.05)
Swain et al. [41] (1, SR only)	USA	Hypercholesterolemia N = 20: 20% males, Age: 30 (23–49) y BMI: NR SBP: 112 mmHg DBP: 68 mmHg	BP secondary crossover randomized double-blind 6 wk 2 arms	High-fiber: 100 g OB in muffins and entrees (21 g/d fiber) Total dietary fiber: 38.9 g/d	Low-fiber: 100 g refined wheat in muffins and entrees Total dietary fiber: 18.4 g/d	No significant change in BP in either group. During high fiber: SBP: 110 mmHg; DBP: 67 mmHg During low fiber: SBP: 107 mmHg; DBP: 65 mmHg Δwt = −0.1, ns
Zhang et al. [42] (2, 3)	China	Hypercholesterolemic N = 166: 39% males Age: 52.7, 53.7 y BMI: 25.5 (0.33) kg/m^2^ SBP: 124.7, 129 mmHg DBP: 80.3, 79.7 mmHg	BP secondary parallel randomized Single-blind 6 wk 2 arms	100 g/d instant oatmeal (~3.6 g/d soluble fiber) Total dietary fiber: 19.3 g/d	100 g/d wheat flour-based noodles Total dietary fiber: 12.9 g/d	SBP: WMD, 0.16 (−2.92, 3.24) DBP: WMD, −0.89 (−3.48, 1.70) Oat: ‡ SBP: 124.7 (1.74) mmHg to 125.7 (1.65) mmHg DBP: 80.3 (1.06) mmHg to 80.1 (0.97) mmHg Control: ‡ SBP: 129.0 (1.78) mmHg to 129.9 (1.69) mmHg DBP: 79.7 (1.09) mmHg to 80.4 (1.00) mmHg No significant difference between groups Δwt = −0.46 kg, +0.67 kg; ns
AlFaris and Ba-Jaber [43]	Saudi Arabia	Type 2 diabetics N = 78: 0% Male Age: 25–60 y BMI: 29.3–36.7 kg/m^2^ SBP: 113.1–132.7 m Hg DBP: 78.5–85.8 mmHg	BP co-primary parallel randomized 3 mo 6 arms Stratified by TG and BMI	Arm 3: 10 g/d OB Arm 4: 10 g/d OB + 5 g/d OO Arm 5: 10 g/d OB Arm 6: 10 g/d OB + 5 g/d OO Included LED + education + meal plans	Arm 1: no intervention Arm 2: LED + education + meal plans	OB + LED results compared to Arm 1: ‡ SBP: High TG, −4.6 (12.0, −3.7; ns) High BMI: −11.9 (12.5, −9.0; *p* < 0.05) DBP: High TG, −3.8 (8.7, −4.8; ns) High BMI: −10.8 (7.6, −12.6; *p* < 0.05) Arm 1 vs. Arm 2 comparisons: ‡ SBP: −1.2 (15.3, −1.0; ns) DBP: −7.7 (7.0, −9.1; *p* < 0.05)
Cicero et al. [44]	Italy	Dyslipidemic subjects N = 83: 42% Male Age: 52.3 (4.4) y wt: 74.5 (17.4) kg Waist: 91.3 (14.6) cm SBP: 128.3 (15.3) mmHg DBP: 81 (9.6) mmHg Fiber: 2.2 (0.9) %en	BP secondary crossover randomized double-blind 8 wk 2 arms	3 g oat β-glucan 10 g sachet	Placebo Oat-based isocaloric 10 g sachet with no β-glucan	Mean changes: SBP: Placebo: −0.6 (−5.4, 4.2; ns) β-glucan: −4.8 (−9.7, 0.1; *p* = 0.053) DBP: Placebo: 0.7 (−2.1, 3.6; ns) β-glucan: −1 (−3.9, 1.9; ns) Δwt (kg): Placebo:‡ −0.2 (−5.4, 5.1; ns) β-glucan: ‡ −0.5 (−6.1, 5.0; ns)
de Souza Leão et al. [45]	Brazil	Metabolic syndrome subjects N = 154: NR % Male Age: 47.6 (12.5) y BMI: 33.9–35 kg/m^2^ SBP: 135.1–135.7 mmHg DBP: 87.3–88.8 mmHg	BP co-primary parallel randomized open-label 6 wk	40 g/d OB (3 g/d β-glucan) + LED	LED	Control: ‡ %HTN from 87.3% to 54.7% (ns) SBP: 136.2 (18.1) to 124.1 (13.7) mmHg (*p* < 0.001) DBP: 87.6 (14) to 80.7 (10.5) mmHg, *p* = 0.002 OB: ‡ %HTN: from 84.3% to 51.9% (ns) SBP: 135.4 (16.9) to 124.6 (17.1) mmHg (*p* < 0.001) DBP: 89.1 to 80.8 (11.4) mmHg (*p* < 0.001) ΔSBP: Control, −12.1; OB, −10.8 (ns) ΔDBP: Control, −6.9; OB, −8.2 (ns) ΔBMI = significant, similar decrease (1.3 kg/m^2^) in both arms (difference ns)

Abbreviations: %en, percentage total energy; AUS, Australia; BMI, body mass index; BP, blood pressure; CI, confidence interval; d, day; DBP, diastolic blood pressure; HTN, hypertension; LED, low-energy diet; LFD, low-fiber diet; LFaD, low-fat diet; MA, meta-analysis; MAP, mean arterial pressure; max, maximum; min, minutes; mo, months; MW, molecular weight; N, sample size; NR, not reported; ns, not significant; NSP, non-starch polysaccharides; OB, oat bran; OO, olive oil; RCT, randomized controlled trial; RTE, ready-to-eat; SBP, systolic blood pressure; SD, standard deviation; SR, systematic review; TG, triglycerides; USA, United States; wks, week; WMD, weighted mean difference; wt, weight; y, years. * Age and BMI were provided as mean (SD) for a population when data was available, or the highest and lowest means for the randomized groups. § Notes whether BP is the primary or secondary outcome in the study. † Data provided as WMD (95% CI) between groups as utilized in MA studies unless otherwise noted. The difference in mean body wt (Δwt) between oat intervention and control group provided over the study, where a positive value indicates a higher wt loss or less wt gain in the intervention group. ‡ Data presented as mean (SD, SEM, %). Data are presented as median (interquartile range) Δ Study referenced in SR/MA: 1. Evans et al. [8]; 2. Khan et al. [25]; 3. Llanaj et al. [24]; 4. Reynolds et al. [28].

## Data Availability

Not applicable.

## Supplementary Materials

The following supporting information can be downloaded at: https://www.mdpi.com/article/10.3390/nu14224772/s1, Table S1: Search Strategy for Systematic Reviews/Meta-Analyses and Key Comprehensive Reviews; Table S2: Search Strategy for Primary Clinical Studies.
