# Supplementary material for: Narrative Review on the Effects of Oat and Sprouted Oat Components on Blood Pressure
Source: Nutrients. 2022 Nov 11;14(22):4772. doi: 10.3390/nu14224772 (PMC9698489; doi:10.3390/nu14224772)
Supplement: Supplementary file 1 [file nutrients-14-04772-s001.zip › nutrients-1981320-supplementary.pdf]

Narrative Review: Effects of Oat and Sprouted Oat Components on Blood Pressure Liska, Dioum, Chu, Mah

Table S1. Search Strategy for Systematic Reviews/ Meta-Analyses and Key Comprehensive Reviews.

Database	Query Terms and Filters	Results	Selected
PubMed<br>4/25/2022	"Avena OR beta-Glucans OR avena sativa OR oat OR oats OR oatmeal* OR oatcake"<br>AND<br>"(blood AND pressure) OR systolic OR diastolic"<br><br>**Filters**: Systematic Review	8 Hits<br>• 3 met criteria for both oats and blood pressure outcome<br>• 5 did not include oats	Llanaj et al. EJN 2022.[1]<br>Khan et al. Nutr Metab Cardiovasc Dis 2018.[2]<br>Evans et al. J Hypertens 2015. [3]
PubMed<br>4/26/2022	"Avena OR oat OR oats OR grain OR cereal"<br>AND<br>"hypertension OR (blood AND pressure) OR systolic OR diastolic"<br><br>**Filters**: Systematic Review	45 Hits<br>• 3 already identified<br>• 2 on wholegrain cereal or RTEC cereal and CVD risk included<br>• 40 did not meet criteria	Kelly et al. Cochrane Database Syst Rev 2007.[4]<br>Priebe and McMonagle. PLoS One 2016.[5] have fulltext
PubMed<br>4/27/2022	"phytonutrient OR phytochemical OR phenolic OR GABA OR (gamma AND aminobutyric)"<br>AND<br>"(blood AND pressure) OR systolic OR diastolic"<br><br>**Filters**: Systematic Review	122 Hits<br>• 8 identified with possibly relevant information to oats<br>• 114 excluded- no relevant information to oats or blood pressure	Sanchez-Martinez et al. Nutrients 2021.[6]<br>Ghaedi et al. Clin Nutr 2020.[7]<br>Godos et al. Antioxidants 2019.[8]<br>Marx et al. Nutrients 2017.[9]<br>Amiot et al. Obes Rev 2016.[10]<br>Rangel-Huerta et al. Nutrients 2015.[11]<br>Kay et al. Mol Nutr Food Res 2012.[12]
PubMed<br>4/27/2022	Search 1: "phytonutrient OR phytochemical OR phenolic OR GABA OR (gamma AND aminobutyric)"<br>AND<br>"(blood AND pressure) OR systolic OR diastolic"<br><br>Search 2: Targeted searches for reviews on oat form (e.g., sprouted)<br><br>**Filters**: Review	>200 Hits<br><br>Selected reviews that included oats or oat components (e.g., phytochemicals) and relevant outcomes	Raguindin et al. Food Chem 2021.[13]<br>Pretorius et al. Metabolites 2021.[14]<br>Aparicio-Garcia et al. Food Chem 2021.[15]<br>Li et al. Food Chem 2020.[16]<br>Ding et al. Food Chem 2019.[17]<br>Cai et al. J Food Sci Technol 2014.[18]<br>Singh et al. Crit Rev Food Sci Nutr 2013.[19]
Cochrane Database<br>5/9/2022	Search 1: Avena OR oats OR oatmeal OR b-glucan OR β-glucan OR beta-glucan OR aminobutyric OR sprouts<br>Search 2: Hypertension OR systolic OR diastolic	Search 1: 42 Hits<br>• Exclude from title search 38<br>• FT review, no oats information<br>• 1 duplicate	Kelly et al. Cochrane Database Syst Rev 2007.[4]

Table S2. Search Strategy for Primary Clinical Studies.

Database	Query Terms and Filters	Results	Fulltext Review
PubMed<br>Search 1<br>5/9/2022	(Avena OR beta-Glucans OR avena sativa OR oat OR oats OR oatmeal* OR oatcake* OR b-glucan* OR (oat AND flavonoid) OR (oat and phenolics) OR (oat AND phytochemicals) OR (oat AND phytonutrient) OR (oat AND aminobutyric) OR (oat AND GABA) OR (oat AND phenolic))<br>**AND**<br>(Hypertension OR (blood AND pressure) OR Systolic OR diastolic OR vascular)<br>**Filters**: Language (English, undetermined)	912 Hits<br>• Excluded duplicates n=8<br>• Excluded not relevant based on title n= 748<br>• Fulltext Review n=156	• No relevant oat or outcome information n=31<br>• Reviews n=27<br>• Animal, in vitro n=42<br>• Select for further review n=56
Google Scholar	(Avena OR sativa OR oat OR oats OR oatmeal OR oatcake (oat AND sprouts) OR beta-glucan) AND (hypertension OR systolic OR diastolic OR (blood pressure)) AND (human OR trial OR clinical) NOT (anticoagulant OR abdomen OR carcinoma) AND English	Hits >973<br>Reviewed first 200. No new studies identified	

KEY REVIEW REFERENCES

1. Llanaj, E.; Dejanovic, G.M.; Valido, E.; Bano, A.; Gamba, M.; Kastrati, L.; Minder, B.; Stojic, S.; Voortman, T.; Marques-Vidal, P.; et al. Effect of oat supplementation interventions on cardiovascular disease risk markers: a systematic review and meta-analysis of randomized controlled trials. *Eur J Nutr* **2022**, doi:10.1007/s00394-021-02763-1.

2. Khan, K.; Jovanovski, E.; Ho, H.V.T.; Marques, A.C.R.; Zurbau, A.; Mejia, S.B.; Sievenpiper, J.L.; Vuksan, V. The effect of viscous soluble fiber on blood pressure: A systematic review and meta-analysis of randomized controlled trials. *Nutr Metab Cardiovasc Dis* **2018**, *28*, 3-13, doi:10.1016/j.numecd.2017.09.007.

3. Evans, C.E.; Greenwood, D.C.; Threapleton, D.E.; Cleghorn, C.L.; Nykjaer, C.; Woodhead, C.E.; Gale, C.P.; Burley, V.J. Effects of dietary fibre type on blood pressure: a systematic review and meta-analysis of randomized controlled trials of healthy individuals. *J Hypertens* **2015**, *33*, 897-911, doi:10.1097/hjh.0000000000000515.

4. Kelly, S.A.; Summerbell, C.D.; Brynes, A.; Whittaker, V.; Frost, G. Wholegrain cereals for coronary heart disease. *Cochrane Database Syst Rev* **2007**, Cd005051, doi:10.1002/14651858.CD005051.pub2.

5. Priebe, M.G.; McMonagle, J.R. Effects of Ready-to-Eat-Cereals on Key Nutritional and Health Outcomes: A Systematic Review. *PLoS One* **2016**, *11*, e0164931, doi:10.1371/journal.pone.0164931.

6. Sánchez-Martínez, L.; Periago, M.J.; García-Alonso, J.; García-Conesa, M.T.; González-Barrio, R. A Systematic Review of the Cardiometabolic Benefits of Plant Products Containing Mixed Phenolics and Polyphenols in Postmenopausal Women: Insufficient

Evidence for Recommendations to This Specific Population. *Nutrients* **2021**, *13*, doi:10.3390/nu13124276.

7. Ghaedi, E.; Foshati, S.; Ziaei, R.; Beigrezaei, S.; Kord-Varkaneh, H.; Ghavami, A.; Miraghajani, M. Effects of phytosterols supplementation on blood pressure: A systematic review and meta-analysis. *Clin Nutr* **2020**, *39*, 2702-2710, doi:10.1016/j.clnu.2019.12.020.
8. Godos, J.; Vitale, M.; Micek, A.; Ray, S.; Martini, D.; Del Rio, D.; Riccardi, G.; Galvano, F.; Grosso, G. Dietary Polyphenol Intake, Blood Pressure, and Hypertension: A Systematic Review and Meta-Analysis of Observational Studies. *Antioxidants (Basel)* **2019**, *8*, doi:10.3390/antiox8060152.
9. Marx, W.; Kelly, J.; Marshall, S.; Nakos, S.; Campbell, K.; Itsiopoulos, C. The Effect of Polyphenol-Rich Interventions on Cardiovascular Risk Factors in Haemodialysis: A Systematic Review and Meta-Analysis. *Nutrients* **2017**, *9*, doi:10.3390/nu9121345.
10. Amiot, M.J.; Riva, C.; Vinet, A. Effects of dietary polyphenols on metabolic syndrome features in humans: a systematic review. *Obes Rev* **2016**, *17*, 573-586, doi:10.1111/obr.12409.
11. Rangel-Huerta, O.D.; Pastor-Villaescusa, B.; Aguilera, C.M.; Gil, A. A Systematic Review of the Efficacy of Bioactive Compounds in Cardiovascular Disease: Phenolic Compounds. *Nutrients* **2015**, *7*, 5177-5216, doi:10.3390/nu7075177.
12. Kay, C.D.; Hooper, L.; Kroon, P.A.; Rimm, E.B.; Cassidy, A. Relative impact of flavonoid composition, dose and structure on vascular function: a systematic review of randomised controlled trials of flavonoid-rich food products. *Mol Nutr Food Res* **2012**, *56*, 1605-1616, doi:10.1002/mnfr.201200363.
13. Raguindin, P.F.; Adam Itodo, O.; Stoyanov, J.; Dejanovic, G.M.; Gamba, M.; Asllanaj, E.; Minder, B.; Bussler, W.; Metzger, B.; Muka, T.; et al. A systematic review of phytochemicals in oat and buckwheat. *Food Chem* **2021**, *338*, 127982, doi:10.1016/j.foodchem.2020.127982.
14. Pretorius, C.J.; Tugizimana, F.; Steenkamp, P.A.; Piater, L.A.; Dubery, I.A. Metabolomics for Biomarker Discovery: Key Signatory Metabolic Profiles for the Identification and Discrimination of Oat Cultivars. *Metabolites* **2021**, *11*, doi:10.3390/metabo11030165.
15. Aparicio-García, N.; Martínez-Villaluenga, C.; Frias, J.; Crespo Perez, L.; Fernández, C.F.; Alba, C.; Rodríguez, J.M.; Peñas, E. A Novel Sprouted Oat Fermented Beverage: Evaluation of Safety and Health Benefits for Celiac Individuals. *Nutrients* **2021**, *13*, doi:10.3390/nu13082522.
16. Li, X.; Wang, P.; Zhu, J.; Yi, J.; Ji, Z.; Kang, Q.; Hao, L.; Huang, J.; Lu, J. Comparative study on the bioactive components and in vitro biological activities of three green seedlings. *Food Chem* **2020**, *321*, 126716, doi:10.1016/j.foodchem.2020.126716.
17. Ding, J.; Johnson, J.; Chu, Y.F.; Feng, H. Enhancement of γ-aminobutyric acid, avenanthramides, and other health-promoting metabolites in germinating oats (Avena sativa L.) treated with and without power ultrasound. *Food Chem* **2019**, *283*, 239-247, doi:10.1016/j.foodchem.2018.12.136.

18. Cai, S.; Gao, F.; Zhang, X.; Wang, O.; Wu, W.; Zhu, S.; Zhang, D.; Zhou, F.; Ji, B. Evaluation of γ- aminobutyric acid, phytate and antioxidant activity of tempeh-like fermented oats (Avena sativa L.) prepared with different filamentous fungi. *J Food Sci Technol* **2014**, *51*, 2544-2551, doi:10.1007/s13197-012-0748-2.

19. Singh, R.; De, S.; Belkheir, A. Avena sativa (Oat), a potential neutraceutical and therapeutic agent: an overview. *Crit Rev Food Sci Nutr* **2013**, *53*, 126-144, doi:10.1080/10408398.2010.526725.
